# Knowledge, attitude and practice GAP in family planning usage: an analysis of selected cities of Uttar Pradesh

**DOI:** 10.1186/s40834-016-0031-4

**Published:** 2016-10-18

**Authors:** Anjali Singh, K. K. Singh, Prashant Verma

**Affiliations:** Department of Statistics, Banaras Hindu University, Varanasi, India

**Keywords:** Contraceptives, Family Planning, Knowledge Attitude and Practice, Chi-Square

## Abstract

**Background:**

The GAP between the knowledge of contraception and its actual practice is well recognized in the literature of family welfare studies. The present study assessed the relation between the level of knowledge and practice of contraception among the women and sought to explore the reasons behind the Knowledge, Attitude, and Practice - GAP (KAP GAP) regarding contraceptive users in six cities of Uttar Pradesh.

**Method:**

Present analysis based on 17,643 currently married women aged 15 to 49. A Bivariate analysis (*χ*2 test) and a multivariable logistic regression were performed for the study.

**Result:**

The highest percentages of respondents (women) were in the age group 35–49 (40–45 %) in all the districts considered. Knowledge of contraceptives was almost universal; tubal ligation and pill were the commonly known methods. Information about the contraceptive methods was mostly obtained through the husband. In the present study, there was a highly significant association (*p* < 0.01) of age group, educational status of respondents, the number of living children, the wealth of the respondent, media exposure and husband’s education with the variable KAP GAP for all six cities. Health concern issues in all the districts were the most prominent reason for not using contraception.

**Conclusion:**

There differences in the socioeconomic and demographic factors exist, which lead to KAP GAP in the family planning (FP) usages. Therefore, in designing effective family planning programme, there is a need to understand the various factors which influence the practice of contraception.

## Background

India became the pioneer nation in the world to launch a nationwide family planning programme in the year 1952, with an intention to reduce fertility and thereby to stabilize the population growth in the near future, even though India is the second most populous country in the world. The family planning programme was initiated with a very cautious approach in India, after the independence (1952) natural method of family planning was considered to be the most appropriate technique for limiting births but a major breakthrough in contraceptive technology occurred, when intrauterine device known as the ‘*Lippes Loop*’ was accepted in India in 1965, the government started promoting the device through intensive campaigns.

In 1977 the programme was renamed as *Family Welfare Programme*. The Family Welfare Programme has relaxed its compulsive approaches in the modification of family planning at various nodes so as to make the programme more educational and wholly voluntary. Desired family size and timing of births are two primary objectives that are fulfilled by the family planning through contraception.

Although the family planning programme was not as successful as was expected, it has succeeded in generating universal knowledge of family planning methods among the masses [1]. But, even with this high awareness of contraception there exists a large gap between the knowledge and Practice of these methods due to the existing variations in the socioeconomic and geographical characteristics within its territory [2].

For measuring the success of Family Planning Programmes there exists one method suggested by a WHO expert in 1975 which is the evaluation of knowledge, attitude, and behavior among people regarding the family planning methods. The concept of KAP - GAP theory was postulated by Bongaarts in 1991, “*The proportion of currently married women who want no more children and are not practicing birth control referred to as the conventional KAP-GAP or unmet need. That is a discrepancy between the practice of contraception and reproductive intentions. This GAP is then assumed to be an indication of unmet need for contraception. Estimates of this measure are readily obtained from most fertility surveys because information on only two items is required: the desire for more children and the current practice of contraception.*” [3]

The concept of KAP-GAP given by Bongaarts and Bruce utilized for understanding the causal factors of contraceptive behavior in Pakistan (Punjab province) [4]. The usage of contraceptive methods has increased over a period but there still exists a KAP- GAP i.e. a GAP between knowledge, attitude, and practices for contraception [5].

Considering the above literature as the base of study, the present research carried out in six major urban districts of Uttar Pradesh, with an objective to assess the knowledge of various family planning methods and also to explore the current trends in usage of contraceptive methods by the women of reproductive age group (15–49), so that the KAP GAP of family planning can be targeted. The most populous state of India, Uttar Pradesh, is the focus state of this study for several reasons. It is the most populated state in India with approximately 16.4 % (more than 19 billion population as of 2011 census) of the total population. Among the high fertility states, Uttar Pradesh with the total fertility rate (TFR) 3.82 is the one that has shown a near stall in fertility decline in the past period. The low contraceptive prevalence rate (47.4) and high level of fertility in Uttar Pradesh are of considerable concern to the Indian Government (NFHS-III (2005–2006)) [6]. This study attempts to explore the reasons for this KAP GAP and the factors affecting the outcome of family planning programme and to have a better understanding of the situation to help the government in the formulation of policies and modify its approach in Uttar Pradesh.

## Methods

### Data source

The data use for the present study was collected by Measurement, Learning & Evaluation (MLE) Project as the part of data collection for the evaluation of Urban Reproductive Health Initiatives, implemented by FHI360 with funding from the Bill & Melinda Gates Foundation. Data collection took place between January and July 2010 in six cities of Uttar Pradesh: Agra (3007), Aligarh (3112), Allahabad (2670), Gorakhpur (3022), Moradabad (2817), and Varanasi (3015). MLE collected information from the representative sample of 17,643 currently married women aged 15–49 years. It is designed to improve family planning and reproductive health services for urban residents and to provide estimates of fertility, mortality, family planning, and health care for urban (slum and non-slum) Uttar Pradesh. The present analysis is based on 17,643 currently married women who were in the reproductive age group 15–49 years (See reference [7] for details of the sampling frame and study design).

### Dependent and explanatory variables

The primary variables of interest (dependent variables) are knowledge, attitude, practice of family planning methods and KAP GAP. The explanatory variables used in the study are age group of women, education level, place of residence, number of living children, religion, caste, economic status, media exposure and husband education. In the present study age of the currently married women has been classified into five groups which are (15–19), (20–24), (25–29), (30–34) and (35–49) years. Education of women has been categorized into four parts namely no education, primary education, secondary education and higher education. Place of residence has been classified into two groups (non-slum and slum). A number of living children have been classified into five groups as no children, one child, two children, three children and four & above children. The religion variable has been classified into two categories as Non-Muslim and Muslim; in the Non-Muslim religion category Hindu, Christian, Sikh, Buddhist/Neo-Buddhist, Jain, and others have been used.

In this study, caste has been taken into three components, namely scheduled caste/scheduled tribe, other backward caste, and others. The description of these caste categories is given as follows The Scheduled Caste (SC) and Scheduled Tribe (ST) are official designations given to various groups of historically disadvantaged indigenous people in India, the second one is Other Backward Caste (OBC) which is a collective term used by the Government of India to classify castes which are socially and educationally disadvantaged and the last one is Others category also known as Forward Class, denote groups of people who do not qualify for any of the positive discrimination schemes operated by the government of India. Wealth has been categories into three parts (Poor, Medium and Rich) Women who fall under the ‘poorest’ and ‘poorer’ wealth index groups are put into the category of ‘poor’ while women who are observed to be in the ‘richer’ and ‘richest’ wealth index groups are considered to be of ‘rich’ in the present study. Media exposure classified into two categories, namely no exposure and media exposure to at least one form of media (newspaper/magazine, listing radio and watching television). Husband’s education level is classified into two categories not educated and educated.

### Statistical methods

Descriptive analysis has been done to explore the levels of knowledge (on different types of contraceptive and knowledge level), attitude and practice among currently married women living in six cities of Uttar Pradesh. To examine the degree and pattern of KAP GAP among women of six cities, a bivariate technique was used and the association between the variables was determined using *χ*2 test. For investigating the important socioeconomic and demographic determinants of KAP GAP univariable and multivariable logistic regression technique was employed. Data was analyzed using SPSS software (version 19).

## Results

Before going into the detailed analysis of the study that how KAP GAP is operating in six cities of Uttar Pradesh, one must have knowledge of the demographic and socioeconomic characteristics of the sample involved under study.

Table 1 corresponds to the percentage distribution of women involved in the study by their different socio-demographic and economic characteristics. It was observed that the highest percentage of respondents were in the age group of 35–49, which corresponded to 40 to 45 % of respondents in the entire city. Nearly 32.3 % of women had received no schooling; 9.8, 34.9 and 22.8 % of respondents have enrolled in primary, secondary and higher education respectively. Further, the percentage of illiterate women was found to be the highest in Aligarh city (42.3 %). About 81.7 % of respondents live in non-slum area, and maximum slum population (26.1 %) lived in Varanasi city. Among the respondents, 30.3 % had parity four or higher than four while 25.5 % had parity two at the time of the survey. Moradabad and Aligarh are predominantly Muslim majority cities whereas, the smallest percentage Muslims were found in Agra (12.8 %). The 42.9 % of the study subjects belonged to other backward class. About 42.8 % of the entire samples of women belonged to rich wealth group, and 36.7 % of women belonged to poor wealth group. Further, the percentage distribution of women in all cities according to the wealth was almost uniform in the entire sample. Around three-quarters of respondents had exposure of at least one form of media; media exposure is highest around 90 % in Allahabad. The educational composition of respondent’s husband comprised that 17 % of the husband had no education.

**Table 1:** Percentage distribution of women according to different characteristics

Background characteristics	Agra	Aligarh	Allahabad	Gorakhpur	Moradabad	Varanasi	Total
Age group							
15–19	3.5	3.6	1.7	2.1	2.2	1.8	2.5
20–24	17.2	15.1	13.7	14.9	12.9	13.7	14.8
25–29	21.6	20.2	20.3	19.3	18.7	21.7	20.6
30–34	18.0	20.0	21.1	19.3	21.0	19.0	19.5
35–49	39.7	41.1	43.2	44.3	45.2	43.9	42.6
Educational status							
No education	38.0	42.3	22.0	26.9	36.0	32.1	32.3
Primary education	9.8	9.2	9.5	8.3	9.3	12.6	9.8
Secondary education	33.9	28.8	35.7	39.3	33.9	38.3	34.9
Higher education	18.2	19.7	32.8	25.5	20.8	16.9	22.8
Place of residence							
Non Slum	75.0	81.3	89.2	90.3	86.8	73.9	81.7
Slum	25.0	18.7	10.8	9.7	13.2	26.1	18.3
No of living children							
No child	8.5	9.1	8.8	10.2	10.1	8.4	9.0
One child	16.7	12.4	17.8	16.2	13.7	15.7	15.8
Two children	24.9	21.1	30.0	25.8	23.3	25.4	25.5
Three children	18.6	19.7	19.2	20.9	20.0	18.4	19.3
Four & more than 4 children	31.1	37.8	24.3	26.9	32.5	32.2	30.3
Religion							
Non muslim	87.2	66.6	81.0	81.5	62.6	77.8	78.5
Muslim	12.8	33.4	19.0	18.5	37.4	22.2	21.5
Caste							
Sc/St	30.8	20.7	15.3	12.7	13.5	12.3	18.5
OBC	32.2	39.3	40.0	47.2	48.8	54.2	42.9
Others	37.0	40.0	44.7	40.0	37.6	33.5	38.6
Wealth index							
Poor	37.5	36.9	32.9	38.4	36.6	38.1	36.7
Medium	19.9	20.5	21.9	19.9	20.0	20.2	20.4
Rich	42.6	42.5	45.2	41.6	43.2	41.7	42.8
Media exposure							
No exposure	19.4	21.4	10.3	11.0	22.1	19.9	27.9
Exposure to at least one form of media	80.6	78.6	89.7	89.0	77.9	80.1	72.1
Husband education							
Not educated	27.1	42.1	27.0	22.8	33.4	22.9	17.0
Educated	72.9	57.9	73.0	77.2	66.6	77.1	83.0

### Knowledge of contraceptives

The underlying section deals with the methods, the source of information and source of services availed by the respondents in six major cities of Uttar Pradesh. The knowledge of contraceptives refers to the number of contraceptive methods known to the women, sources of information for family planning known, and the sources of family planning services known by respondents.

From Table 2, it is clear that knowledge of contraceptives was almost universal in all six urban cities of Uttar Pradesh, where every woman has heard about at least one of the contraceptive methods regardless of educational background and socioeconomic status. All except two women in Varanasi city (more than 99 %) have heard about contraceptives and knew at least one method. Though this study reveals the higher prevalence of knowledge of contraceptives among respondents, the knowledge varied from one method to another also from one city to another. In Moradabad condom (male) was the most popular method known by the respondents, while pill was the most commonly known method in Aligarh and Allahabad, and for rest of the three cities, tubal ligation was the commonly known method. The IUCD is another most popular method cited by respondents, followed by the Vasectomy. However, female condom was the least known method for respondents. Among permanent methods, the knowledge percentage of tubal ligation was much higher than vasectomy in all cities, which indicates that tubal ligation is a well-known family planning method than that of the male sterilization. The analysis shows that Rhythm was a most known method among traditional methods followed by withdrawal method. The knowledge of traditional methods was highest in Agra (rhythm (19.8 %), withdrawal (13.2 %)) and lowest in Aligarh (rhythm (6.4 %), withdrawal (1.8 %)).

**Table 2:** Percentages of different contraceptive methods known by respondents

Method	Agra	Aligarh	Allahabad	Gorakhpur	Moradabad	Varanasi	Total
At least one method	100.0	100.0	100.0	100.0	100.0	99.9	100.0
Modern method							
Pill	63.1	62.0	67.5	54.0	66.9	62.6	63.1
Injectables	16.9	9.6	13.8	16.6	14.9	21.6	16.3
IUCD	32.3	23.6	39.3	26.6	32.0	45.8	35.0
Condom (Male)	59.8	61.3	63.4	62.2	73.1	58.5	62.5
Condom (Female)	0.6	0.2	0.4	0.2	1.6	2.0	0.8
Tubal ligation	69.4	48.1	57.0	68.1	59.8	70.7	64.5
Vasectomy	28.3	6.0	11.1	14.2	10.0	32.7	19.6
Traditional method							
Rhythm	19.8	6.4	9.6	15.4	11.8	15.2	14.1
Withdrawal	13.2	1.8	3.0	5.5	5.3	9.3	7.1
Lam	2.5	0.1	0.1	0.8	0.3	3.3	1.2

The distribution of the source of the knowledge of family planning methods is shown in Table 3. The study suggests that information about the contraceptive methods was mostly obtained through Spousal (husband) communication which was consistent in all six cities of Uttar Pradesh. Media was the second most popular source of information followed by relatives. But in two cities Gorakhpur and Varanasi health personnel were the third most popular source of information in place of relatives, and Allahabad friends/peer educator was the third popular source of information.

**Table 3:** Percentage of sources of family planning information

Source	Agra	Aligarh	Allahabad	Gorakhpur	Moradabad	Varanasi	Total
Health personal	9.6	5.1	8.3	13.1	6.0	13.3	9.2
Partner	62.8	27.5	27.2	51.8	44.1	48.3	43.5
Friend/Peer educator	7.5	11.8	12.4	7.9	10.7	7.4	9.7
Relatives	12.3	13.5	7.3	9.9	11.3	11.2	11
Media	18.7	14.9	21.4	22.7	16.0	21.6	18.9

Table 4 shows that the percentage distribution of knowledge about the places from where the respondents get family planning. The result indicates that about 90.7 % of respondents knew at least one place, where from family planning services can be made available. The pharmacy/drugstore was the most cited source of family planning services in all six cities. Private clinics and public medical sectors were other options from which respondents said that they could receive family planning services.

**Table 4:** Percentage distribution of source of family planning services known by respondents

Knowledge	Agra	Aligarh	Allahabad	Gorakhpur	Moradabad	Varanasi	Total
Any place	89.0	93.7	90.6	89.5	93.8	91.6	90.7
Public medical sector	4.4	13.5	9.7	6.7	12.9	6.5	9.2
Private medical sector	7.7	10.8	9.8	6.7	11.5	8.7	9
Pharmacy/Drug store	67.6	25.7	45.2	55.6	39.9	62.6	48.7

### Practice of family planning method

**Fig. 1:**
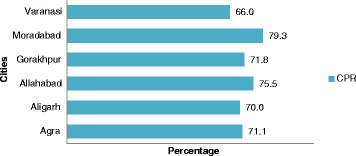
Represent the percentage of Contraceptive Prevalence Rate for all selected cities of Uttar Pradesh (2010)

Figure 1 presents the CPR of currently married women using any family planning method for all six cities. To obtain a true contraceptive use rate, the denominator should reflect the population at risk (of pregnancy), i.e., sexually active women who are not infecund, pregnant, or amenorrheic. The numerator should indicate the number of contraceptive users from that population. The Figure shows that the CPR is highest among currently married women in Moradabad (79.3 %) and lowest in Varanasi (66.0 %), while in all other four cities Agra, Aligarh, Allahabad, and Gorakhpur the practice of family planning methods are figured were nearly comparable to 71.1, 70.0, 75.5 and 71.8 % respectively.

Table 5 represents the percentage distribution of all users according to the method they choose for family planning. Among all available contraceptive methods, tubal ligation was the most commonly used method in four cities Agra (34.5 %), Allahabad (36.4 %), Gorakhpur (38.9 %), and Varanasi (45.5 %) while in Aligarh and Moradabad male condom was the most adopted method of contraception. Respondents also preferred the natural methods and this method was most commonly used in Aligarh (32.8 %) and least often employed in Varanasi (14.8 %). The analysis shows that among permanent methods of contraception, the percentage of using tubal ligation was much higher than male sterilization. The other available modern family planning methods such as combining pill and IUD were the least used methods by the respondents in all six cities.

**Table 5:** Pattern of family planning method usage

Method	Agra	Aligarh	Allahabad	Gorakhpur	Moradabad	Varanasi	Total
Tubal ligation	34.5	22.2	36.4	38.9	28.7	45.5	34.7
Male sterilization	0.1	0.2	0.2	0.2	0.1	0.5	0.2
Natural methods	23.7	32.8	26.4	27.8	21.6	14.8	24.4
Male condom	30.9	33.3	25.9	24.3	42.7	26.6	31
Combined pill	3.2	2.5	1.8	2.5	2.0	3.3	2.4
IUD/loop	2.7	4.3	5.0	2.2	3.3	7.1	4.1

### Knowledge attitude practice gap

Table 6 represents the percentage distribution of KAP GAP among the women according to the socioeconomic and demographic characteristics of all six urban cities of Uttar Pradesh. The highest percentage of KAP GAP was found in Varanasi (34 %) and lowest percentage was found in Moradabad (20.7 %). It observed from the table that the KAP GAP was affected by the socio-economic and demographic differential. In age group (15–19 years) KAP GAP was highest for all cities except Allahabad and it decreased in age group (20–24) (25–29) and (30–34) years except for city Agra (where the KAP GAP again increase in 30–34 age group) and again increases in the 35–49 years age groups in all cities except Allahabad. The association of KAP GAP and education of the women was negative. Not educated women have the greater percentage of KAP GAP while the percentage of KAP GAP was decreasing as the level of education increases, and this result held true for all five cities except Gorakhpur where the second highest percentage of KAP GAP (24.6 %) found in the women of higher education group. KAP GAP is greater in the women who live in a slum area in comparison to the women live in non-slum area. As the number of living children increased KAP GAP decreases up to two children but it again increases for three and more living children, this pattern was followed by all four cities except Gorakhpur and Moradabad. The percentage of KAP GAP was higher in Muslims as compared to non-Muslims (Hindu, Christian, Buddhist and others). Scheduled caste/scheduled tribe women have a higher percentage of KAP GAP followed by others backward class in all four cities except Agra and Aligarh where the highest percentage of KAP GAP found in other backward class. The association of KAP GAP and wealth of the women was also negative, and the scenario was same for all cities. Among the women who had at least one form of media exposure, the percentage of KAP GAP was low in comparison to those who had no exposure to family planning message. For the background variable husband’s education KAP GAP was highest in the no education group in all six selected cities.

**Table 6:** Percentage of KAP GAP according to different characteristics

Background characteristics	Agra	Aligarh	Allahabad	Gorakhpur	Moradabad	Varanasi
Total	28.9	30.4	24.5	28.2	20.7	34.0
Age group						
15–19	47.5	54.5	28.4	54.3	51.9	50.2
20–24	35.5	44.4	37.6	33.2	34.4	44.3
25–29	20.5	29.6	26.6	31.4	19.7	31.5
30–34	27.1	25.7	21.2	17.7	10.5	26.7
35–49	31.8	25.8	18.7	29.2	22.7	35.5
	(*χ*2 = 44.37, *p* = 0.000)	(*χ*2 = 35.83, *p* = 0.000)	(*χ*2 = 42.42, *p* = 0.000)	(*χ*2 = 31.10, *p* = 0.000)	(*χ*2 = 39.92, *p* = 0.000)	(*χ*2 = 28.49, *p* = 0.000)
Educational status						
No education	39.7	41.3	49.4	36.9	29.6	45.6
Primary education	34.3	30.6	20.6	23.8	21.9	36.1
Secondary education	23.6	26.6	19.6	21.2	16.9	33.5
Higher education	18.1	14.5	15.5	24.6	13.6	18.3
	(*χ*2 = 63.51, *p* = 0.000)	(*χ*2 = 100.10, *p* = 0.000)	(*χ*2 = 107.74, *p* = 0.000)	(*χ*2 = 17.45, *p* = 0.001)	(*χ*2 = 43.01, *p* = 0.000)	(*χ*2 = 60.30, *p* = 0.000)
Place of residence						
Non-slum	27.6	29.4	23.3	27.5	19.3	32.6
Slum	33.1	32.3	34.9	35.2	30.2	37.5
	(*χ*2 = 6.35, *p* = 0.007)	(*χ*2 = 0.70, *p* = 0.223)	(*χ*2 = 12.06, *p* = 0.001)	(*χ*2 = 3.64, *p* = 0.037)	(*χ*2 = 7.34, *p* = 0.006)	(*χ*2 = 3.88, *p* = 0.028)
No of living children						
No child	58.6	54.9	45.9	83	66.3	57.2
One child	29.4	32.7	23.1	23.7	22.1	35.6
Two children	24.1	21.4	16.6	24	17.9	25.5
Three children	27.5	27.2	26.3	22.5	15.6	31.9
Four & more than 4 children	33	34.7	37.7	36.8	22.6	40.8
	(*χ*2 = 19.02, *p* = 0.001)	(*χ*2 = 26.39, *p* = 0.000)	(*χ*2 = 52.31, *p* = 0.000)	(*χ*2 = 56.21, *p* = 0.000)	(*χ*2 = 49.73, *p* = 0.000)	(*χ*2 = 48.38, *p* = 0.000)
Religion						
Non muslim	28.1	26.1	22.1	27.3	20.2	29.7
Muslim	33.6	37.5	33.4	31.6	21.4	45.8
	(*χ*2 = 4.06, *p* = 0.044)	(*χ*2 = 11.54, *p* = 0.001)	(*χ*2 = 18.84, *p* = 0.000)	(*χ*2 = 0.25, *p* = 0.613)	(*χ*2 = 1.03, *p* = 0.308)	(*χ*2 = 26.11, *p* = 0.000)
Caste						
Sc/St	32.6	31.9	35.5	31.9	25	42.2
OBC	32.7	35.7	24.3	31.1	22.3	35.8
Others	22.8	23.9	21.4	24	17.4	28.2
	(*χ*2 = 16.95, *p* = 0.000)	(*χ*2 = 15.38, *p* = 0.000)	(*χ*2 = 22.86, *p* = 0.000)	(*χ*2 = 11.91, *p* = 0.003)	(*χ*2 = 8.29, *p* = 0.016)	(*χ*2 = 11.42, *p* = 0.003)
Wealth index						
Poor	35.9	43.5	38.4	34.1	29.2	42.1
Medium	32.7	27.6	27	27.8	23.2	36.6
Rich	21.4	20.3	16.6	24.1	13.4	26
	(*χ*2 = 29.16, *p* = 0.000)	(*χ*2 = 63.13, *p* = 0.000)	(*χ*2 = 69.96, *p* = 0.000)	(*χ*2 = 38.61, *p* = 0.000)	(*χ*2 = 51.16, *p* = 0.000)	(*χ*2 = 42.91, *p* = 0.000)
Media exposure						
No exposure	39.8	42.7	33.7	37	29.1	44.6
Exposure to at least one form of media	25.4	22.1	21.8	25.8	17	31.1
	(*χ*2 = 29.32, *p* = 0.000)	(*χ*2 = 74.44, *p* = 0.000)	(*χ*2 = 28.72, *p* = 0.000)	(*χ*2 = 37.59, *p* = 0.000)	(*χ*2 = 21.78, *p* = 0.000)	(*χ*2 = 32.02, *p* = 0.000)
Husband education						
Not educated	41.7	42.8	45.5	36.8	29.4	43.1
Educated	26.1	27	22.7	27.3	18.5	31.8
	(*χ*2 = 26.26, *p* = 0.000)	(*χ*2 = 21.98, *p* = 0.000)	(*χ*2 = 52.58, *p* = 0.000)	(*χ*2 = 17.48, *p* = 0.000)	(*χ*2 = 26.35, *p* = 0.000)	(*χ*2 = 19.30, *p* = 0.000)

### Chi-square analysis

In the present study, there was a highly significant association (*p* < 0.01) among age group, educational status of respondents, the number of living children, the wealth of the respondent, media exposure, and husband’s education with the variable KAP GAP for all six cities. But for the religion variable the association was highly significant (*p* < 0.01) for three cities Aligarh, Allahabad and Varanasi and significant (*p* < 0.05) only for Agra. Similarly, an association of the variable place of residence with KAP GAP was not significant only for Aligarh. The relationship between caste and KAP GAP variable was highly significant for all five cities except Moradabad where the significance level was less than 0.05. The value of chi-square with its level of significance (*p*-value) for all the variable shown in Table 6.

### Logistic regression analysis

To estimate the degree of effect of each independent variable on the study variable and to verify whether the differences provided by the different categories of the various explanatory variables considered in our study are statistically significant or not, we have applied logistic regression technique and obtained crude as well as adjusted odds ratios.

Table 7 depicts the crude and adjusted odds ratios with their confidence interval of KAP GAP among women for different socio-economic and demographic characteristics, obtained from logistic regression analysis. From the figures of crude odds ratio of KAP GAP one can conclude that place of residence, religion, the caste of women and husband education were highly significant but as soon as the procedure controlled for all explanatory variables the differences arises from these variables remain no more significant.

**Table 7:** Logistic regression results of KAP GAP by different background characteristics

Background characteristics	Crude odds ratio	Confidence interval (95 %)		Adjusted odds ratio	Confidence interval (95 %)	
		Lower	Upper		Lower	Upper
Age group						
15–19	Ref			Ref		
20–24	0.627^**^	0.463	0.848	0.989^*^	0.767	1.47
25–29	0.368^**^	0.273	0.497	0.734^*^	0.526	1.023
30–34	0.273**	0.202	0.369	0.573^**^	0.406	0.809
35–49	0.379**	0.282	0.509	0.766^*^	0.542	1.082
Educational status						
No education	Ref			Ref		
Primary education	0.614^**^	0.521	0.724	0.683^**^	0.575	0.812
Secondary education	0.529^**^	0.475	0.588	0.688^**^	0.602	0.788
Higher education	0.324^**^	0.286	0.367	0.499^**^	0.417	0.597
Place of residence						
Non Slum	Ref			Ref		
Slum	1.435**	1.283	1.606	1.134	1.007	1.276
No of living children						
No child	Ref			Ref		
One child	0.247^**^	0.191	0.32	0.247^**^	0.188	0.323
Two children	0.178^**^	0.138	0.229	0.205^**^	0.155	0.269
Three children	0.214^**^	0.165	0.279	0.206^**^	0.153	0.277
Four & more than 4 children	0.328^**^	0.255	0.422	0.231^**^	0.171	0.314
Religion						
Non Muslim	Ref			Ref		
Muslim	1.415^**^	1.285	1.559	1.106	0.986	1.239
Caste						
Sc/St	Ref			Ref		
OBC	0.906^**^	0.801	1.023	0.972	0.847	1.115
Others	0.608^**^	0.536	0.691	0.967	0.834	1.121
Wealth index						
Poor	Ref			Ref		
Medium	0.688^**^	0.611	0.776	0.900^**^	0.79	1.024
Rich	0.425^**^	0.384	0.469	0.680^**^	0.597	0.774
Media exposure						
No Exposure	Ref			Ref		
Exposure to atleast one form of Media	0.514^**^	0.467	0.565	0.725^**^	0.652	0.807
Husband education						
Not educated	Ref			Ref		
Educated	0.531^**^	0.474	0.594	1.051	0.919	1.202

* *p* < 0.05

** *p* < 0.01

Taking 15–19 years age group as a reference category, the odds for KAP GAP for the women with increasing age was decreased up to age 34 and again increased in the age group 35–49. Media exposure significantly affects the KAP GAP among the women. The odds for women who were exposed to at least one form of media were lower as compared to the women who had no exposure to any type of media. In the logistic regression model, one more covariate education level of the women was also included. Keeping no education group as a reference category primary and secondary education groups had similar odds which were lower than reference category.

Further considering the no of living children, as the no of living children increased adjusted odds for KAP GAP decreased up to parity two and again increases for three and higher parity these results were highly significant. This study also indicates that Wealth index significantly influenced the KAP GAP with raising the level of wealth index there was a rapid decline in the odds ratio.

### Reasons for KAP GAP

Table 8 shows that decision for adopting any family planning method was influenced by health concern issues (20.7 %); this is one of the primary reasons for the KAP GAP. Evidently for Agra and Varanasi, the same reason accounted for 28.9 %, and 27.9 % women were not using any contraceptive method. The study show that some of the respondents had not planned their fertility and leave it up to God, so this is also a second important reason for KAP GAP in all cities except Varanasi where fear of side effects was a second important reason for not using contraception. The KAP GAP due to the reason of disapproval of husband and other relatives was subtle (3.2 %) in this study.

**Table 8:** Reason given for not using any contraceptive methods

Reasons	Agra	Aligarh	Allahabad	Gorakhpur	Moradabad	Varanasi	Total
Fear of side effect	18.5	9.4	6.7	11.2	9.8	23.4	14.7
Health concern issue	28.9	11.2	10.3	22.1	9.4	27.9	20.7
Up to god	24.1	14.3	9.5	17.6	22.1	19.7	18.2
Disapproval of husband/ relatives	2.5	3.8	2.1	2.3	2.6	6.0	3.2

## Discussion

### Finding and interpretation

The above study reveals that knowledge among married women about at least one family planning method was universal, and it was independent of their socioeconomic characteristic [8, 9]. Substantial variation in the knowledge of usages of one contraceptive method over another was clearly visible in the study and is well supported by the existing literature. Among permanent method knowledge about tubal ligation was far better than vasectomy [10, 11]. The variation of knowledge was also visible for cities like in Aligarh and Allahabad pill was the most known method and it was not same for all other cities. The analysis shows that spouse communication was the major source of knowledge carrier regarding family planning usage. The role of health personals in the awareness programme of family planning was not found to be satisfactory in our results. Knowledge about the source to get family planning was also excellent, and a medical store was the most prominent source for getting family planning services.

The present analysis shows that more than half of the respondents use family planning methods in all cities. Tubal ligation and male condom were most used methods in our study. The reasons for adoption of tubal ligation may be because of its permanent nature, physical safety and comfort and no fear of failure [10].

The Majority of women have the knowledge of family planning in spite of only one-third of study population not practicing any method. This difference shows that there exists KAP GAP in the population among these urban districts of Uttar Pradesh. KAP GAP was found to be affected by the socioeconomic differentials; maximum KAP GAP was discovered in the females of the younger age group (15–19) years [12] and it was negatively associated with respondent education, it means uneducated women had higher KAP GAP as compared to educated women. Media exposure was also an important factor in shaping the decision of women to use family planning [2, 13–15] this may be a reason that media exposure affects KAP GAP. Instead of these covariates total no of children and women’s wealth index were also an important factors which influences the KAP GAP but husband education was not significantly associated with KAP GAP after adjusting the regression for other background characteristics. A decision to adopt family planning was influenced by issues of health concerns [10, 11, 16].

### Strength and weakness

The MLE survey is the recent study that covers Family planning issues in a comprehensive manner in Uttar Pradesh, the most populous state in India. KAP GAP in six major districts in Uttar Pradesh gives an indication of a universal presence of knowledge but differences in the actual practices of family planning methods based on recent data from the year 2010. The studied population did not reflect the whole population of Uttar Pradesh it covered only urban part of the state which is the biggest limitation of the study.

### Unanswered question and future research

Every study forms a basis for further research and in the line of a survey carried out; we have witnessed differences in knowledge and practice regarding usage of family planning methods leading to KAP GAP in six major cities including slum and non-slum population of Uttar Pradesh. Contraceptive method generally divides into two groups, modern and traditional contraception the above study address the GAP but the strength or magnitude of KAP GAP according to the type of contraceptive method and its corresponding differentials have not been dealt with in the study because of limitation of the survey, also the factors that are encouraging women to use family planning were not analyzed in the paper which needs to be addressed in further research.

## Conclusion

The analysis explicitly mentioned that there exist differences in the socioeconomic and demographic factors, which in turn affects contraceptive usages, leading to KAP GAP in the family planning. The research clearly brings out the reason according to the region specific primary correlates for KAP GAP. Therefore, in designing effective family planning programmes, policy makers must understand the various factors which influence the practice of family planning methods according to regions. It comes from the study that method related issues are also a major reason for not using the contraceptive, so family planning programme should have to focus on giving the correct information and knowledge for the different methods to improve the family planning practices.

## Abbreviations

CPR: Contraceptive prevalence rate
FP: Family planning
KAP: Knowledge attitude and practice
MLE: Measurement learning & evaluation
OBC: Other backward caste
Sc/St: Scheduled caste/Scheduled tribe
